# The Interdependence of Long- and Short-Term Components in Unmasked Repetition Priming: An Indication of Shared Resources

**DOI:** 10.1371/journal.pone.0144747

**Published:** 2015-12-11

**Authors:** Matt R. Merema, Craig P. Speelman

**Affiliations:** School of Psychology and Social Science, Edith Cowan University, Joondalup, Western Australia, Australia; University of Leicester, UNITED KINGDOM

## Abstract

It has been suggested that unmasked repetition priming is composed of distinct long-and short-term priming components. The current study sought to clarify the relationship between these components by examining the relationship between them. A total of 60 people (45 females, 15 males) participated in a computer-based lexical decision task designed to measure levels of short-term priming across different levels of long-term priming. The results revealed an interdependent relationship between the two components, whereby an increase in long-term priming prompted a decrease in short-term priming. Both long-term and short-term priming were accurately captured by a single power function over seven minutes post repetition, suggesting the two components may draw on the same resources. This interdependence between long- and short-term priming may serve to improve fluency in reading.

## Introduction

When a word is recognised the brain is primed in preparation for interacting with that same word more efficiently should they appear again in future [1]. This process, referred to as repetition priming, is a memory phenomenon distinct from explicit memory recall [2, 3, 4] and is thought to decay slowly over a period of months [5]. Research into the decay of unmasked repetition priming has suggested that this stable, long-term priming effect observed by Grant and Logan [5] might be preceded by a distinctly different short-term priming event [6, 7, 8, 9]. This short-term priming event is different to that observed in masked priming paradigms [10] and presents as temporally similar to working memory in explicit recall. McKone and Dennis [11] propose that this implicit short-term memory for words may be present to assist with integrating successive items, such as is required when processing sentences.

McKone [6], for example, demonstrated that a substantially larger but shorter-lived priming effect occurs following the repetition of an unmasked word within 10 seconds. The decay rate of repetition priming found in experiment 1 of McKone [6] was accurately captured by an exponential function (P = 93.5e^-0.63 L^ + 49.1, where P equals the amount of priming in milliseconds and L represents the number of intervening items). This function accounted for 98% of the variance in priming decay, suggesting that priming for unmasked words decayed to 63% of their value for each intervening item presented between the prime and target (i.e., every two seconds) to a minimum value of 49.1*ms*. The Helmert contrasts conducted by McKone comparing priming at various lags identified no further decay after an eight second (3 intervening items) delay. Consequently, the data was interpreted as partial support for the idea that an initial short-term priming effect is superimposed on a smaller but longer lasting priming value of 49.1*ms*.

In addition, McKone [6] identified that non-words decay almost immediately to a long-term baseline level, dissociating the long- and short-term components. The study also found that word-frequency affects long-term priming but not short-term priming. Subsequent dissociations observed between long- and short-term priming have also been utilized as support for separate components in unmasked repetition priming. For example, McKone and Dennis [11] dissociated the long- and short-term components in unmasked repetition priming by demonstrating that the slower decay of words than non-words in long-term priming was evident in stimuli presented both visually and aurally. For short-term priming, however, the word/non-word dissociation was only evident in visually-presented stimuli. McKone and Dennis [11] suggest this reflects differing needs of the orthographic and phonological systems, whereby aurally-presented non-words remain primed for longer out of a need to treat spoken non-words as potential words.

Whilst these studies provide support for the existence of separate long- and short-term components in unmasked repetition priming, the extent to which the two components draw on separate or shared resources is still relatively unclear. In the long- and short-term components of explicit memory, there is evidence to suggest that the two are positively associated with one another. In explicit memory tasks, long-term memory typically improves short term recall [12, 13]. For example, Hulme et al. [12] found memory span to be significantly better for stimuli with a pre-existing representation in long-term memory (i.e., words) than for stimuli with no pre-existing representation (i.e., non-words). However, the nature of the relationship between the long- and short-term components of implicit memory is still unknown.

Despite evidence for a positive association between the long- and short-term components of explicit memory, there is evidence for the reverse with implicit memory. The word-frequency effect, whereby less priming is observed with higher frequency words in repetition priming (e.g., [3, 14, 15, 16, 17]), implies greater familiarity (and therefore presumably stronger representation in long-term memory) results in a smaller contribution from the short-term component. Thus, the long-term (pre-existing) and short-term representations may be negatively associated with one another in implicit memory, suggesting they draw on shared resources. To test this relationship more directly, however, short-term priming could be observed over varying levels of long-term priming.

Given the effect of word frequency, an increase in long-term priming should be accompanied by a decrease in short-term priming. Such a relationship would suggest a sharing of resources between the two components. Thus, if short-term priming decreases consistently as a negative function of long-term priming, it would suggest at least a partial overlap of resources. If, however, short-term priming is unaffected by or positively associated with changes in long-term priming, then one could assume the two components draw on more distinct sets of resources. To examine these possibilities, the current study examined whether or not short-term priming differs under varying levels of long-term priming.

## Method

### Subjects

Sixty people participated in the study (45 females, 15 males), including 43 undergraduate students from Edith Cowan University, Joondalup (35 females, 8 males) and 17 members of the general public (10 females, 7 males). All participants spoke fluent English and had normal or corrected-to-normal vision.

### Ethics Statement

Ethics approval for this research was obtained from the Edith Cowan University Human Research Ethics Committee. Subjects gave written consent to participate in the research.

### Design

A lexical decision task utilizing a one-way repeated measures design was used to assess the effect of varying levels of word repetition throughout the task on long- and short-term priming. Words and non-words were presented in a random order throughout 16 blocks of trials (with an additional practice block at the beginning to enable the participant to gain an understanding of the task). Target words were presented 1, 4, 8 or 16 times throughout the task, allowing a comparison of three different levels of word repetition to assess for differences in long-term priming (note the 1× presentation condition does not provide a measure of long-term priming and is utilized only in the follow-up analysis when mapping decay over varied delays). New words were also presented in each block.

In order to be sensitive to practice effects, long-term priming was measured as the percentage of decrease in mean reaction time (RT) to make the lexical decision for target words in the 4×, 8× and 16× conditions on the first presentation within the final block in comparison to new words presented in the final block. (Mean RTs and response accuracy, and priming values, for all cells are provided in S1 Appendix. Raw data are available in S1 Dataset.) To measure short-term priming, target words in the 4×, 8× and 16× conditions were presented twice consecutively in the final block. Short-term priming was measured as the percentage of decrease in mean RT to identify the lexicality of target words between the first and second presentation in the final block. Whilst it would be preferable to measure long-term priming and short-term priming using the same method (i.e., observing the difference between repetitions), differing lags across the 4×, 8× and 16× conditions meant the extent to which practice effects confound priming values would not be consistent across the different conditions. That is, there would be a much greater confounding to words infrequently repeated, such as in the 4x condition, than to words repeated more often, such as in the 16x condition).

To prevent participants from assuming that all target words would be presented consecutively in the final block, three ‘control’ conditions were created. Apart from not being repeated in the final block, words in the three control conditions were presented in an identical fashion to words in the 4×, 8× and 16× conditions (a control condition was not required for the 1× condition since these words were presented only in the final block). Words from all seven ‘multiple presentations’ conditions (1×, 4×, 8×, 16×, 4 control, 8 control, and 16 control) were retrieved from the same source and were not noticeably distinguishable in any manner. Words allocated to the 1×, 4×, 8× and 16× conditions were rotated through each condition across participants in order to control for possible effects related to individual words. Apart from testing for the speed-accuracy trade-off, non-words and words in the three control conditions were not included in any part of the analysis. The mean time and item delay between word repetitions in the 4×, 8× and 16× conditions are presented in Table 1.

**Table 1:** Mean Time and Item Delay (with Standard Deviation) Between Repeated Words in the 4×, 8× and 16× Conditions.

	Condition		
	16×	8×	4×
Time Delay (seconds)	107.18 (40.93)	215.34 (86.74)	429.43 (197.59)
Item Delay	84.41 (1.10)	170.85 (2.52)	343.74 (6.30)

### Materials

The stimuli used in the task included four-to-seven letter words and non-words. All words had a frequency count of one per million, as ranked by Kucera and Francis [18]. Ten words were allocated to each of the 1×, 4×, 8× and 16× conditions (40 in total), 10 were allocated to each of three control conditions (30 in total) and 20 were allocated to the practice block and to each of the subsequent 16 blocks throughout the task to be presented as new words (340 in total). Non-words were generated by changing one letter of real words to create a pronounceable and orthographically legal letter-string. None of the non-words were generated from stimuli presented as words in the task. The number of non-words presented in each block was equal to half of the total words presented in that same block (475 in total). Stimulus presentation and response recording was controlled by an Apple PowerMacintosh G4 computer with SuperLab software.

### Procedure

Participants were tested individually in a single session lasting approximately 30–40 minutes. For each trial, the participant was required to decide whether the letter-string on the screen was a word (“m” key on the computer keyboard marked “word”) or a non-word (“c” key marked “non-word”). Letter strings were presented in 26pt Arial font and remained on the screen until the participant responded. There was no inter-trial interval, such that each word or non-word was presented immediately following the participant’s response on the previous trial. Handedness was not taken into account as no comparison in the analysis was made between words and non-words. Participants were instructed to respond as quickly and as accurately as possible. No feedback was provided to the participant regarding accuracy or speed throughout the task. Each participant completed a total of 1425 trials.

## Results

The mean RT of one participant and the overall error rate of a second participant fell well outside 2.5 standard deviations above the mean and as a result all data contributed by these two participants were excluded from the analysis. Reaction times on individual trials were excluded if an incorrect response was provided. In addition trials were excluded if they were above 1200*ms* or below 300*ms*. The mean percentage of correct trials excluded was 2.60%. The mean RT and error rate for all words across participants were 761*ms* and 8.44%. The relationship between mean response accuracy and mean RTs for non-words was not significant, *r* ^2^ = 0.003, *p* > .05, suggesting no evidence of a speed-accuracy trade-off. In order to be sensitive to baseline RTs, both long- and short-term priming data was analysed using percentages (i.e., as a proportion of the initial baseline RT).

### Long-Term Priming

A one-way repeated measures analysis of variance (ANOVA) was conducted to determine the effect of number of word presentations (4×, 8× or 16×) on long-term priming. Mauchly’s test indicated that the assumption of sphericity had been violated (χ^2^(2) = 10.30, *p* < .05). Consequently, Greenhouse-Geisser estimates of sphericity were used (*ε* = .86). As anticipated, the results indicated that the percentage of long-term priming increased significantly with the number of presentations throughout the task, *F*(1.71, 97.60) = 4.82, *p* < .05. Bonferroni-adjusted post-hoc tests revealed that the obtained percentage of long-term priming was significantly greater for words presented 16 times (*M* = 18.48%, *SD* = 17.00) than for words presented 4 times (*M* = 10.79%, *SD* = 20.63). However, long-term priming in words presented 8 times (*M* = 15.53%, *SD* = 15.13) was not significantly different to words presented 4 or 16 times.

### Short-Term Priming

A second one-way repeated measures ANOVA was performed to determine the effect of number of presentations on short-term priming. The results indicated that the obtained percentage of short-term priming decreased significantly with number of presentations throughout the task, *F*(2, 114) = 3.65, *p* < .05. Bonferroni-adjusted post-hoc tests revealed that the short-term priming percentage was significantly larger for words presented 4 times (*M* = 23.52%, *SD* = 10.52) than for words presented 16 times (*M* = 18.93%, *SD* = 11.61). However, as with long-term priming, short-term priming for words presented 8 times (*M* = 21.28%, *SD* = 11.90) was not significantly different to short-term priming for words presented 4 or 16 times.

### Rate of Decay

Given the previous analysis suggests long- and short-term priming respond in the opposite manner to increases in word repetition (i.e., all significant increases in long-term priming were accompanied by significant decreases in short-term priming), further analysis was conducted to examine whether both the short and long-term components of repetition priming could be captured accurately by a single decay function. Studies have indicated that long-term decay can be accurately captured by a power function (e.g., [5]), but whether or not a single function can accurately capture both long and short-term decay has not been examined.

To examine the fit of a power function to data that includes both long- and short-term components of repetition priming, mean percentages of priming were plotted for the second presentation of words within the 1×, 4×, 8× and 16× conditions. Percentages of priming were again used in order to be sensitive to baseline RTs. For this analysis, we used priming values in the 1x condition as a measure of short-term priming, given the second presentation immediately followed the first. The 4x, 8x and 16x conditions were used as measures of long-term priming, given their lengthier delays between the first and second presentation of target words in these conditions. The mean time and item delay as well as the mean priming percentage produced between the first and second presentation within each condition is presented in Table 2.

**Table 2:** Mean Time Delay, Item Delay and Percentage of Priming (with Standard Deviation) within each Condition.

	Condition			
	1×	16×	8×	4×
Time Delay (seconds)	0.91 (0.02)	108.45 (4.01)	220.29 (7.18)	418.29 (12.94)
Item Delay	1.00 (0.00)	76.91 (1.07)	165.00 (1.70)	331.93 (1.94)
Priming (%)	26.86 (14.31)	12.50 (14.05)	11.19 (13.31)	10.27 (15.39)
Priming (%) Predicted by Power Functions				
Time Delay	26.81	12.60	11.26	10.18
Item Delay	26.67	12.80	11.25	10.00

The power functions that provided the best fit for decay over time delay (seconds) and decay over item delay were: (Time delay) y = 26.41 × (d^-.158^) and (Item delay) y = 26.67 × (i^-.169^), whereby y equals the percentage of priming, 26.41 and 26.67 equal the constants (the initial, maximum percentage of priming), d represents delay (in seconds), i represents the item delay (where immediate repetition = 1), and -.158 and -.169 equal the unstandardized coefficients for time and item delay, respectively. Based on each set of four means, the models were able to account for 100% (for time delay) and 99.8% (for item delay) of the variance in priming decay (see Table 2).

## Discussion

The current set of results suggest (1) that the long- and short-term components of repetition priming are negatively interdependent and (2) that the decay of both long and short-term components over time (and items) can be captured accurately by a single power function with no enduring baseline. The first set of analyses augment previous research into long- and short-term repetition priming by suggesting the two components have at least a partial overlap of resources (given their negative interdependence). The results of the power function analysis performed subsequently suggest that, although separate long- and short-term components may exist, their decay rates can be accurately captured via a single function. This function analysis also extends the findings of Grant and Logan [5] by demonstrating that, in addition to the loss of priming that occurs between 5 minutes and 2 months post-presentation, the loss of repetition priming that occurs between 1 second and approximately 7 minutes post-presentation can also be captured accurately by a power function.

Long-term priming was found to increase gradually but steadily over 4, 8 and 16 presentations and all changes in priming were in the expected direction. Whilst significant differences were only identified between the 4× and 16× presentation conditions, the non-significant differences are likely a power issue attributable to the random presentation of words within each block (which would have increased the standard error and reduced the power of the ANOVAs). On the contrary, short-term priming decreased gradually but steadily over 4, 8 and 16 presentations. The non-significant differences between the 4× and 8×, and 8× and 16× presentation conditions are also likely attributable to the reduced power brought about by random order word presentation.

On these results alone, the data tends to suggest that the long and short-term components are substitutive and perhaps better described as a single process (i.e., one that simply describes overall priming decay rather than incorporating separate long and short-term components). Indeed, the results may simply be reflective of increasing familiarity through repetition (gains in long-term priming) being accompanied by smaller gains in familiarity (declines in short-term priming).

In the context of McKone and Dennis’s [11] interpretation with regard to sentence processing, this interdependence would help to maintain consistency in word identification between words of varying familiarity. When less familiar words are encountered during reading, they receive a comparatively large short-term priming gain to compensate for lower long-term priming levels. However, when more familiar words are encountered, they require less short-term benefit, since long-term priming levels are already comparatively high. Such a system would allow for more consistency in the speed at which highly familiar and unfamiliar words can be recognised, allowing for more consistent and predictable saccadic eye movements (which already slow the speed of reading, see [19]) and thus greater reading fluency.

One could argue that the results of the initial analyses are not necessarily surprising and that they simply reflect a decreasing increment in priming with each additional word presentation. Thus, on account of a floor effect in repetition priming (each word can only be primed so much), the amount of “space” left for short-term priming as the amount of long-term priming moves closer to its maximum will of course become less and less. Given the various dissociations identified between the two components in previous research, the negative interdependence between long and short-term repetition priming may reflect a sharing of resources between them. The result is interesting in the context of implicit versus explicit memory, since research into the long and short-term components of explicit memory has demonstrated a positively interdependent relationship. The result complements other studies that have identified functional differences between implicit and explicit forms of memory (e.g., [20]).

The accuracy with which the power functions can be fitted to decay after the first presentation within each condition suggests repetition priming deteriorates at a very consistent rate following the word presentation over approximately the first seven minutes (or 330 items). That is, the rate at which the percentage of priming decayed between 1 and 108 seconds post-presentation was the same as the rate of decay between 3 minutes-40 seconds and 7 minutes. Such a result suggests power functions, which are suitable for mapping decay of repetition priming over long periods, are also suitable for predicting decay over shorter periods.

It is worth noting, however, that the results obtained in the current study might be dependent on the lexical decision task. It is possible that with tasks that rely less on familiarity (such as speeded word naming), the same interdependence might not emerge. It would be worth examining this relationship via other tasks to see whether the results of the current study hold up under different paradigms.

For the current study, the negative interdependence between long and short-term repetition priming and the accuracy with which decay over long and short periods can be plotted under the same power function suggests that, whilst long and short-term components of repetition priming can be isolated through various dissociations, their contributions to an overall priming effect are largely substitutive. Both components can be discriminated between; however the contribution of one component to the overall repetition priming effect may be entirely dependent on the amount contributed by the other.

In summary, the current results revealed a negative interdependence between the long- and short-term components of repetition priming and show that overall priming levels decayed consistently (with no fixed baseline level) as a power function of time delay (in seconds) and item delay over the first seven minutes or 330 items post-presentation. It is suggested that the long- and short-term components may be substitutive in their contribution to an overall repetition priming effect, which likely reflects the sharing of a single set of resources. This relationship is reversed to that observed in recall tasks where explicit memory is utilized and may serve to enhance fluency in reading.

## Supporting Information

S1 Appendix Mean Reaction Times (*ms*) and Response Accuracy (%) across Participants (Table A). Mean Priming Values (*ms*) by Priming Type and Condition across Participants (Table B). (DOCX)

S1 Dataset Raw RT and Accuracy data. (XLSX)
