# Supplementary material for: The Interdependence of Long- and Short-Term Components in Unmasked Repetition Priming: An Indication of Shared Resources
Source: PLoS One. 2015 Dec 11;10(12):e0144747. doi: 10.1371/journal.pone.0144747 (PMC4684243; doi:10.1371/journal.pone.0144747)
Supplement: S1 Appendix — (DOCX) [file pone.0144747.s001.docx]

S1 Appendix

Table A. Mean Reaction Times (*ms*) and Response Accuracy (%) across Participants

| Mean Accuracy (%) | Mean Response Times (*ms*) | | |
| --- | --- | --- | --- |
|  | Overall | Words | Non Words |
| 91.56% | 761 | 732 | 831 |

Table B. Mean Priming Values (*ms*) by Priming Type and Condition across Participants

| Long-Term Priming (*ms*)^*^ | | | Short-Term Priming (*ms*)^**^ | | | |
| --- | --- | --- | --- | --- | --- | --- |
| 4x | 8x | 16x | 1x | 4x | 8x | 16x |
| 23 | 43 | 73 | 208 | 156 | 134 | 110 |

^*^Long-term priming was measured as the mean decrease in response time (*ms*) to correctly identify target words in the 4x, 8x and 16x conditions on the first presentation within the final block in comparison to new words in the final block.

^**^Short-term priming was measured as the mean decrease in response time (*ms*) to correctly identify target words between first and second presentation in the final block.
